# Diseases Admitted under the Care of the Physicians of the Westminster Hospital, from the 18th of Dec. 1799, to the 18th of Jan. 1800

**Published:** 1800-02

**Authors:** 


*Diseases admitted under the care of the Physicians of the Westminster Hospital, from the* 18*th of Dec.* 1799, *to the* 18*th of Jan.* 1800.

| | | | |
|---|---|---|---|
| Fevers - - | 7 | Epilepsy - - - | 2 |
| Pleurisy - - - | 3 | Hæmoptoe - - | 2 |
| Scarlatina - - | 2 | Hæmatemesis - - | 1 |
| Amenorrhœa - - | 5 | Itch - - - | 4 |
| Anasarca - - | 4 | Lepra Græcorum - | 1 |
| Ascites - - - | 2 | Lumbago - - | 1 |
| Asthenia - - | 2 | Phthisis - - | 2 |
| Asthma - - | 9 | Paralysis - - - | 2 |
| Catarrh - - | 2 | Rheumatism - - | 8 |
| Cholera - - - | 1 | Struma - - - | 1 |
| Cough - - - | 16 | Tinea - - | 1 |
| Diarrhœa - - | 7 | Vertigo - - - | 1 |
| Dyspepsia - - | 4 | Urticaria - - | 2 |
| Dysuria - - | 2 | Worms - - - | 2 |

Pectoral complaints have not only been uncommonly frequent during the last month, but in *elderly people* especially, they have been attended with untractable symptoms. The practitioner has often found it difficult to decide how far venæsection and the antiphlogistic plan could be usefully persisted in; and in many instances, as often happens in severe winters, the disease has baffled all his attempts to relieve it.

